# Immunostaining of thymidylate synthase and p53 for predicting chemoresistance to S-1/cisplatin in gastric cancer

**DOI:** 10.1038/sj.bjc.6603546

**Published:** 2007-01-09

**Authors:** S Kamoshida, M Suzuki, R Shimomura, Y Sakurai, Y Komori, I Uyama, Y Tsutsumi

**Affiliations:** 1 Department of Pathology, Fujita Health University School of Medicine, Toyoake, Aichi 470-1192, Japan; 2 Department of Surgery, Fujita Health University School of Medicine, Toyoake, Aichi 470-1192, Japan

**Keywords:** thymidylate synthase, p53, S-1, cisplatin, gastric cancer

## Abstract

High expression of thymidylate synthase (TS) and inactivation of p53 are allegedly associated with chemoresistance. The authors evaluated TS and p53 expression in gastric cancer treated with neoadjuvant S-1/cisplatin chemotherapy. Paraffin sections of pretreatment biopsy and surgical specimens from 41 gastric cancers were immunostained for TS and p53 protein after appropriate antigen retrieval. Fifty-one cases without neoadjuvant chemotherapy were also studied. In the pretreatment biopsies, high expression of TS was seen in 8% of the histologic responders, in 28% of the nonresponders and in 31% of the controls. High expression of p53 was observed in 56% of the nonresponders, but in 8% of the responders and in 29% of the controls (*P*<0.01 and *P*<0.05, respectively). The TS- and/or p53-high phenotype was seen in 76% of the nonresponders and in 54% of the controls, but in 8% of the responders (*P*<0.0001 and *P*<0.005, respectively). The data of the surgical specimens were consistent with those of the pretreatment biopsies. These results suggest that immunostaining for TS and p53 protein is useful for pretreatment selection of gastric cancer patients unresponsive to S-1/cisplatin chemotherapy.

Gastric cancer is declining in incidence, but it remains a significant cause of mortality (Parkin *et al*, 1988). Resectability is one of the most important prognostic factors in treating gastric cancer (Yeh and Cheng, 2004). Even in resectable tumours, the recurrence rate is significantly high. For downstaging and lowering the rate of postoperative recurrence, neoadjuvant chemotherapy for gastric cancer has been performed in the high-risk group (Yonemura *et al*, 1996; Yeh and Cheng, 2004). It has been reported that histological grading is a useful prognostic indicator in gastric cancer treated with neoadjuvant chemotherapy (Yonemura *et al*, 1996).

Fluoropyrimidines (5-fluorouracil (5-FU) and its prodrugs) are most widely used for the treatment of solid tumours including gastric cancer. Several kinds of fluoropyrimidines are now in use. S-1 is a novel fluoropyrimidine consisting of tegafur, 5-chloro-2, 4-dihydroxypyrimidine (CDHP, a strong inhibitor of dihydropyrimidine dehydrogenase, 5-FU-catabolizing enzyme) and potassium oxonate with a molar ratio of 1 : 0.4 : 1 (Malet-Martino and Martino, 2002). S-1, especially in combination with cisplatin, shows a promising effectiveness with acceptable toxicities against gastric, pancreatic and non-small-cell lung cancers (Schoffski, 2004; Yoshida *et al*, 2005).

Analysis of the predictors for identifying patients who will be sensitive or resistant to chemotherapeutic agents is one of the approaches for individualizing the treatment. The appropriate decision for chemotherapy leads to the avoidance of unpleasant side effects. At the present time, however, there are no clinically accepted markers that can accurately predict the sensitivity or resistance of gastric cancer against chemotherapeutic agents.

Thymidylate synthase (TS) is the target enzyme of 5-FU, and the inhibition of TS activity is the principal mechanism of its cytotoxicity. Plural preclinical studies have suggested that high TS levels are closely associated with the 5-FU resistance of cancer cells (Beck *et al*, 1994; Peters *et al*, 1994). Investigators have reported the clinical relevance of high *TS* mRNA as a predictor of the resistance to 5-FU/cisplatin or S-1 in gastric cancer (Lenz *et al*, 1996; Ichikawa *et al*, 2004). However, the relationship between immunohistochemical expression of TS in pretreatment biopsy specimens of gastric cancer and the response to fluoropyrimidine-based chemotherapy has revealed conflicting results. Some investigators have shown that the TS expression in gastric cancer is of limited value in predicting the clinical response to fluoropyrimidine-based chemotherapy (Boku *et al*, 1998; Miyamoto *et al*, 2000). In contrast, there is a report demonstrating the clinical relevance of high TS expression as a predictor of the resistance to fluoropyrimidine-based chemotherapy and the short survival of the patients (Yeh *et al*, 1998).

The product of the tumour-suppressor gene, *p53*, is a pleiotropic molecule with plurifunctions. p53 protein normally functions as either a repair initiator of the damaged sense DNA or a trigger of the apoptotic pathway if a sufficient level of damage takes place within the cell (Leonard *et al*, 1995; Harwood *et al*, 1996). It has been demonstrated that p53-dependent apoptosis is associated with the cytotoxic effects of cisplatin or 5-FU plus cisplatin for gastric cancer cells (Ikeguchi *et al*, 1997; Satomi *et al*, 2002; Matsuhashi *et al*, 2004). The absence of wild-type p53 function results in the cellular resistance to anticancer agents such as 5-FU and cisplatin in several cell lines, including gastric cancer (Nabeya *et al*, 1995; Harris, 1996). In addition, several investigators have demonstrated the correlation between the immunohistochemical overexpression of p53 in primary gastric cancer and the resistance to cisplatin-contained regimens (Hamada *et al*, 1996; Cascinu *et al*, 1998; Nakata *et al*, 1998).

There is evidence that TS forms a ribonucleoprotein complex with *p53* mRNA (Liu *et al*, 2002). It is thereby suggested that TS may play a critical role in regulating the cell cycle and the process of apoptosis through its regulatory effects on the expression of p53. In the present study, we immunohistochemically analysed the relationship between the expression of TS and p53 and the effects of neoadjuvant S-1/cisplatin chemotherapy for gastric cancers. We demonstrate here that TS- and/or p53-high expression can be a relevant and useful predictor in determining S-1/cisplatin resistance of gastric cancer.

## MATERIALS AND METHODS

### Patients and specimens

Table 1 summarizes pretreatment clinicopathological features of 92 patients included in the present study. All the patients were treated for advanced gastric cancer between 2001 and 2006 at the Department of Surgery, Fujita Health University Hospital, Toyoake, Japan. Forty-one patients were preoperatively treated with the combination of S-1 (80–120 mg m^−2^ per day for 4 weeks) and cisplatin (35 mg m^−2^ on day 8). One cycle of this regimen was completed in 6 weeks and the next cycle of this regimen was started after 2 weeks off of S-1 administration since the last day of the S-1 administration. At least two cycles of this regimen were performed, depending on the clinical status of these patients. The median of this regimen was consequently two cycles. We have adopted the dose of 35 mg m^−2^ on day 8 of cisplatin, because of the fact that S-1 alone and/or S-1 plus low-dose cisplatin must have caused a significant clinical effect. We have experienced cases showing a significant clinical effect after S-1 alone and/or S-1 plus low-dose cisplatin in neoadjuvant settings (Yoshida *et al*, 2005). Fifty-one patients who did not receive neoadjuvant chemotherapy were also analysed as controls. The two groups were closely matched and there were no differences in the clinicopathological backgrounds. The present study was approved by the institutional ethical review board for human investigation at Fujita Health University.

Pretreatment biopsy specimens and surgically resected tumours were routinely fixed in 10% formalin and embedded in paraffin wax. Sections, 3 *μ*m thick, were cut and mounted on aminopropyltriethoxysilane slides, and stained with haematoxylin and eosin (HE), in order to assess histopathological features and the responsiveness to neoadjuvant chemotherapy under a light microscope.

### Histological assessment of chemotherapeutic effects

Chemotherapeutic effects were histologically evaluated using the surgical specimens, according to the Japanese Classification of Gastric Carcinoma (Japanese Research Society for Gastric Cancer, 1999). Major grading (grades 0–3) and additional minor grading for grade 1 (grades 1a and b) were used, based upon the degree of necrosis or disappearance of tumour cells in the lesion; grade 0: no change, grade 1: mild change (grade 1a: necrosis or disappearance of the tumour seen in less than 1/3 of the entire cancer area, which is regarded as undistinguishable from spontaneous tumour necrosis; and grade 1b: necrosis or disappearance of the tumour seen in more than 1/3 but less than 2/3 of the entire cancer area), grade 2: moderate change (necrosis or disappearance of the tumour seen in more than 2/3 of the entire cancer area, but still with remaining viable tumour cells) and grade 3: severe change (no viable tumour cells remain). The patients with grades 1b and 2 were categorised as the histologic responders, and the patients with grades 0 and 1a as the histologic nonresponders. No grade 3 case was observed.

### Immunohistochemistry

In addition to pretreatment biopsy specimens, one or two representative paraffin blocks of the resected tumour were chosen for immunohistochemical analysis. The sections were deparaffinised with xylene and rehydrated with graded ethanols. Endogenous peroxidase was inactivated by 0.03% hydrogen peroxide in methanol for 30 min. Heat-induced epitope retrieval was applied using a pressure cooker (Delicio 6L; T-FAL, Rumily, France) for 10 min. Optimal soaking solutions, determined by preliminary experiments (Kamoshida *et al*, 2003a, 2003b), were selected for the respective markers: 1 mM ethylenediaminetetraacetic acid solution, pH 8.0 for TS and 10 mM citrate buffer, pH 7.0 for p53. After pressure-cooking, the sections were left at room temperature for cooling in the soaking solution for 30 min.

The sections were then incubated with the primary antibody that reacts specifically with TS (rabbit polyclonal, 1 : 200 dilution; Taiho Pharmaceutical Co., Tokushima, Japan) or p53 (mouse monoclonal, clone DO-7, 1 : 200 dilution; Dako Co., CA, USA), overnight at room temperature. Histofine Simple Stain MAX-PO (Nichirei, Tokyo, Japan), employing the universal immunoperoxidase polymer method, was utilised as a second-layer reagent. The reaction products were visualised in 50 mg dl^−1^ 3,3′-diaminobenzidine tetrahydrochloride solution containing 0.003% hydrogen peroxide. The nuclei were lightly counterstained with Mayer's haematoxylin. Negative control studies were performed without applying the primary antibodies. Sections known to be stained positively were included in each run as positive staining controls.

### Evaluation of immunostaining

The HE-stained and immunostained sections were independently reviewed by two investigators (SK and MS), without prior knowledge of clinical data of the patients. Thymidylate synthase expression was classified into two groups in a semiquantitative manner: ‘low’ expression (negative or positive in 50% or less of tumour cells) *vs* ‘high’ expression (positive in more than 50% of tumour cells). Most of the other investigators defined TS expression of 25–30% as a threshold level (Yeh *et al*, 1998; Miyamoto *et al*, 2000). The reason for the difference is explained as follows: (1) to the best of our belief, TS staining method employed in our study is very sensitive, suitable and reproducible (Kamoshida *et al*, 2003a); (2) in our previous studies using colorectal cancer specimens, 30% was defined as a threshold level of TS expression (Kamoshida *et al*, 2003b, 2004). It has been reported that the mean *TS* mRNA level in gastric cancers is 1.6- to 1.7-fold higher than that in colorectal cancers, and the *TS* mRNA level is closely correlated with immunohistochemical expression of TS (Johnston *et al*, 1995; Uchida *et al*, 2001).

The pattern of immunohistochemical expression of p53 protein, the proportion of p53-positive cells, must be very important in discussing the relationship between p53 protein expression and gene mutation (Hall and Lane, 1994). The occurrence of occasional positive cells does not seem to correlate with obvious abnormality of *p53* gene, but the positive staining in the majority of cells is frequently associated with gene mutation (Baas *et al*, 1994; Shiao *et al*, 2000). For p53, thus, negative or positive staining in 70% or less of tumour cells was considered ‘low’ expression, and positive staining in more than 70% of tumour cells was ‘high’ expression. According to the principle of imunohistochemical evaluation using multiple clinical samples, the intensity of staining should not be considered for the judgement of positivity (Kamoshida *et al*, 2004).

The evaluation of biopsy specimens was regarded appropriate when two or more biopsy pieces were available, as reported previously (Kamoshida *et al*, 2003b).

### Statistical analysis

The Fisher's exact probability test was employed for determining the statistical significance of correlations between the marker expression and histological chemotherapeutic effects. *P*-values <0.05 were considered statistically significant.

## RESULTS

### Marker expression

Thymidylate synthase immunoreactivity was observed in the cytoplasm of cancer cells. p53 protein was invariably localized in the nuclei. Little difference in the staining pattern of TS and p53 was seen between invasive and noninvasive components within the same tumours. Epithelial cells located in the generative zone of normal gastric mucosa and in the basal half of intestinal metaplastic mucosa were consistently immunoreactive for TS. In addition, TS was expressed in such nonepithelial cells as germinal centre lymphocytes, plasma cells, endothelial cells, fibroblasts and smooth muscle cells. No apparent p53 expression was detected in the non-neoplastic tissue and cells.

### Chemotherapeutic effects and marker expression in pretreatment biopsy specimens

Histological chemotherapeutic responders consisted of 15 (37%) out of 41 cases (grade 1b: six cases and grade 2: nine cases), and the remaining 26 (63%) were categorised as the nonresponders (grade 0: eight cases and grade 1a: 18 cases).

Table 2 shows the relationship between chemotherapeutic effects and marker expression in the pretreatment biopsy specimens. Adequate biopsy material obtained from two cancerous parts or more was available in 38 out of the 41 cases receiving neoadjuvant chemotherapy and in 48 out of the 51 control untreated cases. High TS expression was observed in one lesion (8%) of 13 responders, in seven lesions (28%) of 25 nonresponders and in 15 lesions (31%) of 48 control untreated cases: no significant differences were noted.

High expression of p53 was observed in 14 lesions (56%) of the nonresponders and in 14 lesions (29%) of the control cases, but in one lesion (8%) of the responders: there was significant difference between the responders and the nonresponders (*P*<0.01), and between the nonresponders and the control cases (*P*<0.05). Accuracy of the p53 expression for predicting chemoresistance was 68%; 26 (12 responders and 14 nonresponders) out of 38 patients treated with neoadjuvant chemotherapy.

**Figure 1:**
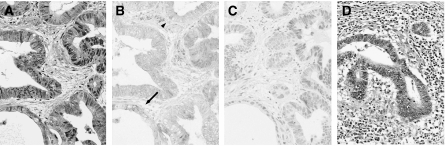
The pretreatment biopsy specimen (**A**–**C**) and the resected tumour (**D**) from a representative gastric cancer, which responded to neoadjuvant S-1/cisplatin chemotherapy. (**A**, **D**): HE staining, (**B**): TS immunoreactivity, (**C**): p53 immunoreactivity. Chemotherapy-induced histological changes including the disintegration of glandular structures with marked inflammatory cell infiltration is noted in the resected tumour when compared with the pretreatment biopsy specimen. The cytoplasm of only a few cancer cells (arrow) and plasma cells (arrowhead) in the stroma are positive for TS. There are no p53-positive cells.

**Figure 2:**
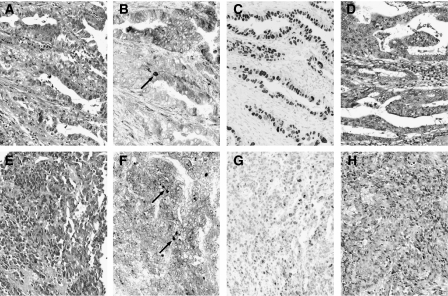
The pretreatment biopsy specimen (**A**–**C**, **E**–**G**) and the resected tumour (**D**, **H**) from two representative gastric cancers, which did not respond to neoadjuvant S-1/cisplatin chemotherapy. (**A**, **D**, **E**, **H**): Haematoxylin and eosin staining, (**B**, **F**): TS immunoreactivity, (**C**, **G**): p53 immunoreactivity. Little histological changes are discernible after chemotherapy. In a case shown in the top panels (**A**–**D**), a number of cancer cells express both TS and p53. In another case shown in the bottom panels (**E**–**H**), TS is expressed in a number of cancer cells. p53-positive nuclei are heterogeneously observed. Plasma cells in the stroma (arrows) are also immunoreactive for TS.

The TS- and/or p53-high phenotype was demonstrated in 19 lesions (76%) of the nonresponders and 26 lesions (54%) of the control cases, but in one lesion (8%) of the responders: the differences between the responders and the nonresponders or the control cases were statistically significant (*P*<0.0001 and *P*<0.005, respectively). Accuracy of the combination of TS and p53 expression for predicting chemoresistance was 82%; 31 (12 responders and 19 nonresponders) out of 38 patients treated with neoadjuvant chemotherapy. Representative immunostaining patterns in the pretreatment biopsy specimens are shown in Figures 1 and 2.

### Chemotherapeutic effects and marker expression in surgically resected tumours

Table 3 shows the relationship between chemotherapeutic effects and marker expression in the surgical specimens. The data of the surgical specimens were consistent with those of the pretreatment biopsies. High expression of TS was seen in one lesion (7%) of 15 responders, in seven lesions (27%) of 26 nonresponders and in 14 lesions (27%) of 51 control untreated cases: no significant differences were detected. High expression of p53 was detected in 14 lesions (54%) of the nonresponders, but in one lesion (7%) of the responders and in 15 lesions (29%) of the control cases: The statistical significance was noted between the responders and the nonresponders (*P*<0.005), and between the nonresponders and the control cases (*P*<0.05). The TS- and/or p53-high phenotype was observed in 19 lesions (73%) of the nonresponders and 26 lesions (51%) of the control cases, but in one lesion (7%) of the responders: The differences between the responders and the nonresponders or the control cases were statistically significant (*P*<0.0001 and *P*<0.005, respectively).

### Correlation of TS and p53 expression between pretreatment biopsy specimens and surgically resected materials

Table 4 demonstrates the concordance rate of TS and p53 expression between the pretreatment biopsy specimens and the resected materials. Thymidylate synthase expression was concordant in 34 (89%) out of 38 cases treated with neoadjuvant chemotherapy and in 41 (85%) out of 48 control untreated cases. The concordance of p53 expression was noted in 37 (97%) of the cases treated with neoadjuvant chemotherapy and in all of the control cases.

## DISCUSSION

To our knowledge, the present study is an initial attempt to predict the effects of S-1/cisplatin chemotherapy by the combination of TS and p53 immunostaining in gastric cancer. Thymidylate synthase, a critical target for fluoropyrimidines, catalyses the methylation of deoxyuridine monophosphate to deoxythymidine monophosphate, an essential step in DNA biosynthesis (Malet-Martino and Martino, 2002). When the pretreatment biopsy specimens were analysed, high expression of TS was observed in 8% of the responders and in 28% of the nonresponders, without significant difference. Yeh *et al* (1998) reported that high expression of TS significantly predicted the resistance to high dose 5-FU and leucovorin and the short survival of the patients, but others failed to demonstrate the significant relationship between the TS expression and the effects of fluoropyrimidine-based chemotherapy (Boku *et al*, 1998; Miyamoto *et al*, 2000). These results suggest that the effects of fluoropyrimidine-based chemotherapy for gastric cancers are unable to be predicted by TS alone.

p53 participates in apoptotic pathways following treatment with DNA-damaging agents such as cisplatin (Ikeguchi *et al*, 1997; Satomi *et al*, 2002; Matsuhashi *et al*, 2004), and inactivation of the *p53* gene contributes to the resistance to anticancer agents in several cell lines, including gastric cancer (Nabeya *et al*, 1995; Harris, 1996). Fifteen years ago, a series of important issues were raised concerning the immunohistochemical assessment of p53 protein (Wynford-Thomas, 1992). Of particular note was that immunohistochemically detected p53 protein expression did not necessarily indicate the presence of *p53* genetic mutations. The pattern of immunohistochemical expression of p53 protein, the proportion of p53-positive cells, should be crucial in discussing the relationship between p53 immunoreactivity and genetic mutation (Hall and Lane, 1994). For example, focal (heterogeneous) expression, the occurrence of dispersed positive cells, does not seem to correlate with the obvious abnormality of *p53* gene (Baas *et al*, 1994; Shiao *et al*, 2000). It may rather reflect an accumulation of wild-type p53 protein as a result of either a response to DNA damage, alterations in the normal degradation process, or the stabilization of the gene product by an interaction with viral or cellular proteins (Baas *et al*, 1994; Kirsch and Kastan 1998). In contrast, diffuse (homogeneous) expression, the positive staining in the majority of cells, is frequently associated with mutation (Baas *et al*, 1994; Shiao *et al*, 2000). This view is supported by the clinical data, in which the differences in expression patterns are significantly correlated with the patient's prognosis (Barnes *et al*, 1993; Shiao *et al*, 2000). In addition, the intensity of staining should not be considered for the judgment of staining results, according to the principle of immunohistochemical evaluation using multiple clinical samples (Kamoshida *et al*, 2004). Still, it remains possible that there might be some discordance between p53 immunoreactivity and the genetic abnormality: for example, the presence of missing deletions may result in the abolishment of protein production, and some point mutations may lead to the production of unstable proteins without an increase of the half-life (Wynford-Thomas, 1992).

In the present study, p53 expression was classified into two groups: low expression (negative or nuclear positivity in ⩽70% of tumour cells) *vs* high expression (nuclear positivity in >70% of tumour cells). High expression of p53 in the pretreatment biopsy specimen was observed in 56% of the nonresponders but in 8% of the responders (*P*<0.01). These results let us postulate that the high expression of p53 protein in our series may reflect the diffuse (homogenous) overexpression of the mutant-type protein, leading to the resistance to S-1/cisplatin chemotherapy. However, this must be confirmed by additional study using molecular assays, such as single-strand conformational polymorphism analysis and direct sequencing.

When we analysed the combination of TS and p53 expression in the pretreatment biopsy, TS- and/or p53-high phenotype was seen in 76% of the nonresponders but in 8% of the responders (*P*<0.0001). Accuracy predicting the chemoresistance to S-1/cisplatin was 82% when p53 expression was combined with TS expression, whereas it was 68% when only p53 expression was applied. These results indicate that the TS- and/or p53-high phenotype as determined by immunohistochemistry is a strong predictor of the resistance to S-1/cisplatin chemotherapy. Reportedly, TS forms a ribonucleoprotein complex with the *p53* mRNA and the functional consequence of the binding is translational repression (Liu *et al*, 2002). In the lesions showing p53-low but TS-high phenotype (observed in five lesions of the nonresponders but in none of the responders), thus, TS may play a critical role in regulating the process of apoptosis through its regulatory effects on expression of p53.

We demonstrated acceptable consistency in TS and p53 expression between the pretreatment biopsy specimens and surgically resected materials. The results suggest that the expression of TS and p53 protein is hardly changed after S-1/cisplatin chemotherapy, and immunostaining of TS and p53 in pretreatment biopsy specimens can be utilised for predicting the chemoresistance to S-1/cisplatin. Discrepant results of TS were probably due to the heterogenous distribution seen in most cancer tissues. *In vitro* study has demonstrated that twice the amount of TS is observed in gastric cancer cells after continuous exposure to 5-FU, when compared to the untreated cells (Yukimoto *et al*, 2001). However, the TS induction after drug exposure has not been documented in gastric cancer patients undergoing fluoropyrimidine-based chemotherapy (Uchida *et al*, 2001). A similar discrepancy between *in vitro* and *in vivo* systems has found in colorectal cancer (Peters *et al*, 2000; Uchida *et al*, 2001; Kamoshida *et al*, 2003b).

In conclusion, we demonstrated that high expression of TS and/or p53 in the pretreatment biopsy specimens predicted the chemoresistance to S-1 plus cisplatin in gastric cancer. We believe that the data of the present retrospective study provide with the basis for a prospective study, in which immunohistochemical expression of TS and p53 is utilised as a weapon for tailor-made chemotherapy.

## Figures and Tables

**Table 1:** Pretreatment clinicopathological features of 41 patients treated with neoadjuvant chemotherapy and 51 control patients

**Clinicopathological features**	**Patients treated with neoadjuvant chemotherapy (*n*=41)**	**Control patients (*n*=51)**
*Age (years)*		
<60	15	17
⩾60	26	34
		
*Gender*		
Male	27	33
Female	14	18
		
*Primary site*		
Cardia	11	12
Fundus	20	26
Antrum	10	13
		
*Histologic type* a		
Intestinal	16	19
Diffuse	22	29

a Pretreatment biopsy specimens were available for analysis in 38 out of 41 patients with neoadjuvant chemotherapy, and 48 out of 51 control patients without neoadjuvant chemotherapy.

**Table 2:** Relationship between expression of TS and p53, and effects of neoadjuvant S-1/cisplatin chemotherapy in pretreatment biopsy specimens

**Marker expression**	Histological responders (%)^*^ (*n*=13)^a^	Histological non-responders (%)^**^ (*n*=25)^a^	Control untreated cases (%)^***^ (*n*=48)^a^	***P*-value**		
				^*^ *vs* ^**^	^*^ *vs* ^***^	^**^ *vs* ^***^
*TS*						
High	1 (8)	7 (28)	15 (31)	0.22	0.15	>0.99
Low	12 (92)	18 (72)	33 (69)			
						
*p53*						
High	1 (8)	14 (56)	14 (29)	<0.01^b^	0.16	<0.05^b^
Low	12 (92)	11 (44)	34 (71)			
TS- and/or p53-high	1 (8)	19 (76)	26 (54)	<0.0001^b^	<0.005^b^	0.08
TS- and p53-low	12 (92)	6 (24)	22 (46)			

a Pretreatment biopsy specimens were available for analysis in 38 out of 41 patients with neoadjuvant chemotherapy, and 48 out of 51 control patients without neoadjuvant chemotherapy.

b Statistically significant.

^*^Responders, ^**^Non-responders, ^***^Control cases.

**Table 3:** Relationship between expression of TS and p53, and effects of neoadjuvant S-1/cisplatin chemotherapy in surgically resected tumours

**Marker expression**	Histological responders (%)^*^ (*n*=15)	Histological non-responders (%)^**^ (*n*=26)	Control untreated cases (%)^***^ (*n*=51)	***P*-value**		
				^*^ *vs* ^**^	^*^ *vs* ^***^	^**^ *vs* ^***^
*TS*						
High	1 (7)	7 (27)	14 (27)	0.22	0.16	>0.99
Low	14 (93)	19 (73)	37 (73)			
						
*p53*						
High	1 (7)	14 (54)	15 (29)	<0.005^a^	0.09	<0.05^a^
Low	14 (93)	12 (46)	36 (71)			
TS- and/or p53-high	1 (7)	19 (73)	26 (51)	<0.0001^a^	<0.005^a^	0.09
TS- and p53-low	14 (93)	7 (27)	25 (49)			

a Statistically significant.

^*^Responders, ^**^Non-responders, ^***^Control cases.

**Table 4:** Correlation of TS and p53 expression between pretreatment biopsy specimens and surgically resected materials

	**Resected tumour**		
**Pretreatment biopsy**	**High**	**Low**	**Concordance (%)**
Cases treated with neoadjuvant chemotherapy (*n*=38)			
*TS*			
High	6	2	34 (89%)
Low	2	28	
			
*p53*			
High	14	1	37 (97%)
Low	0	23	
			
Control untreated cases (*n*=48)			
*TS*			
High	11	4	41 (85%)
Low	3	30	
			
*p53*			
High	14	0	48 (100%)
Low	0	34	
